# Disentangling the effects of species interactions and environmental factors on the spatial pattern and coexistence of two congeneric *Pinus* species in a transitional climatic zone

**DOI:** 10.1002/ece3.9275

**Published:** 2022-09-11

**Authors:** Zhichun Yang, Ying Luo, Nan Ye, Lishunan Yang, Qiulong Yin, Shihong Jia, Chunmei He, Zuoqiang Yuan, Zhanqing Hao, Arshad Ali

**Affiliations:** ^1^ School of Ecology and Environment Northwestern Polytechnical University Shaanxi China; ^2^ CAS Key Laboratory of Forest Ecology and Management, Institute of Applied Ecology Chinese Academy of Sciences Liaoning China; ^3^ Forest Ecology Research Group, College of Life Sciences Hebei University Baoding Hebei China

**Keywords:** density dependence, different life‐history stages, environmental factors, *Pinus* congeneric species, spatial pattern and associations, transitional climatic zone

## Abstract

Congeneric species are critical for understanding the underlying ecological mechanisms of biodiversity maintenance. Ecological mechanisms such as conspecific negative density dependence, species differences in life‐history stages related to habitat preference, and limiting similarity are known to influence plant fitness, thereby influencing species coexistence and biodiversity. However, our understanding of these phenomena as they apply to coexistence among coniferous species is limited. We studied two congeneric *Pinus* species, *Pinus armandii* (PA) and *Pinus tabulaeformis* (PT), both of which are common pioneer species typically succeeded by oaks (*Quercus*), in a 25‐ha warm temperate deciduous broad‐leaved forest. Here, we addressed the following questions: (1) How do population structures and distributions patterns of these two *Pinus* species vary with respect to different life‐history stages? (2) Does intra‐ and interspecific competition vary with respect to three life‐history stages? And (3) What are the relative contributions of topographic and soil variables to the spatial distributions of the species across the three life‐history stages? In addressing these questions, we utilized the pair‐correlation function g(r), redundancy analysis (RDA), variance partitioning (VP), and hierarchical partitioning (HP) to identify habitat preferences and conspecific negative density dependence at different life‐history stages from small to large trees. The results revealed that in both *Pinus* species, individuals in different life‐history stages were subject to significant habitat heterogeneity, with a tendency for small trees to be distributed at higher latitudes that may be represents climate‐change‐driven migration in both species. In addition, the effects of conspecific negative density dependence on PT were stronger than those on PA due to limited dispersal in PT. Furthermore, we found that interspecific competition was weak due to the species differences in resource utilization and preference for key habitats. Our study shows that congeneric *Pinus* species avoids competition by exploiting distinct habitats and provides insight into forest community structure.

## Abstract 2

The results revealed that in both Pinus species, individuals in different life‐history stages were subject to significant habitat heterogeneity, with a tendency for small trees to be distributed at higher latitudes that may be represents climate‐change driven migration in both species. In addition, the effects of conspecific negative density dependence on PT were stronger than those on PA due to limited dispersal in PT. Furthermore, we found that interspecific competition was weak due to the species differences in resource utilization and preference for key habitats.

## 1 INTRODUCTION

Biodiversity is fundamental to the functioning of Earth (Xue, 2021), especially in forest ecosystems (Lykhovyd, 2021), and provides numerous ecosystem services essential for biological and social development (Patil et al., 2021). In recent decades, climate change has severely affected biodiversity and associated ecological processes (Sintayehu, 2018). However, climate change can alter the distributions of tree species and cause significant declines in biodiversity and ecosystem functions as tree species shift in distribution toward higher latitudes and altitudes (Khalatbari Limaki et al., 2021). The study of dominant congeneric species is a well‐established approach for revealing the ecological mechanisms underlying biodiversity maintenance in complex ecosystems (Losos, 2010; Vleminckx et al., 2018) because congeners have similar tolerance limits for similar environmental conditions due to their close phylogenetic relationship (Darwin, 1864).

However, whether the similarity of congeneric species contributes to their coexistence has long been debated (Li et al., 2014; Mooney et al., 2008; Yamada et al., 2005). Considerable evidence supports the notion that species coexistence is maintained by multiple coexistence mechanisms, as predicted by niche theory, neutral theory, and hypotheses of habitat preference and conspecific negative density dependence (Burns & Strauss, 2011; Chesson, 2000b; Macarthur & Levins, 1967). Niche theory (Kraft et al., 2015) predicts that closely related species compete more intensely than distantly related species due to their requirements for similar resources. Moreover, survival under resource‐limited conditions may prevent the coexistence of closely related species (Mooney et al., 2008; Ribeiro et al., 2021; Simberloff, 1970), as reported for *Quercus* species (Yuan et al., 2018) and *Lauraceae* species (Li et al., 2014). Although highly similar species may delay competitive exclusion through nearly neutral dynamics, species differences are ultimately required for stable coexistence (Chesson, 2000a; Levine & Hillerislambers, 2009). However, closely related species can co‐occur in similar environments as a result of shared traits (Valiente‐Banuet & Verdú, 2007); that is, the existence of habitat preferences produces spatial niches, which strongly stabilizes the coexistence of two species (Pigolotti & Cencini, 2010). Previous studies have predicted that closely related species are functionally similar and are thus expected to share similar habitats (Harvey & Pagel, 1991), as reported for *Neolistea* (Yamasaki et al., 2013) and *Acer* (Zhang et al., 2010) species. According to the hypothesis of conspecific negative density dependence, species coexistence can be promoted if intraspecific competition is stronger than interspecific (Chesson, 2000a; Janzen, 1970). In addition, some studies have suggested that heterogeneity in resource availability is important for the coexistence of congeneric species (Tanaka et al., 2008). Many congeners coexist in most biodiverse tropical forest communities and low‐biodiversity temperate forest communities (Tanaka et al., 2008; Yamada et al., 2005; Zhang et al., 2010). However, some studies have found that the competition between congeneric species is not strong (Li et al., 2014; Sedio et al., 2012; Yang et al., 2018), and raising, the question could be how can similar congenerics coexist?

Previous studies have suggested that the coexistence of local species is maintained through microhabitat and species interactions (Inman‐Narahari et al., 2014). Both environmental and biotic interactions structure congeneric species coexistence; in tree species, their spatial patterns and interactions are limited by various biological factors and environmental conditions in the community (Erfanifard & Stereńczak, 2017; Liao et al., 2015; Liu et al., 2014). Among the biotic processes influencing coexistence, dispersal ability and interactions at early life‐history stages are the most important. For instance, wind disperse‐seeded species to disperse distance shorter than gravity and animals secondary disperse‐seeded species (Thomson et al., 2011). *Pinus tabulaeformis* (PT) relies on wind for seed dispersal, which is facilitated by its small seeds with attached wings (Greene & Johnson, 1993), whereas *Pinus armandii* (PA) depends on animals for seed dispersal (Chang et al., 2012). Limited seed dispersial plays an important role in determining the spatial patterns of adult plants (Burns & Pugnaire, 2005; Normand et al., 2011). Negative density‐dependent processes (Janzen, 1970) lead to lower survival and germination of seeds that fall close to the parent plant. In addition, soil resource variation influences the distributions of many individual species (Baldeck et al., 2013), and topography influences water availability and the soil nutrient ratio (Yuan et al., 2019). Additionally, Jara‐Guerrero et al. (2015) found that in Ecuadorian tropical dry forests, the distributions of most species were generally affected by spatial heterogeneity rather than dispersal ability. The spatial patterns of species are usually explained by a combination of dispersal limitation, habitat filtering, and species interactions (Zhou et al., 2019). Thus, analysis of the spatial patterns of congeneric species and the influencing factors offers unique opportunities and presents challenges for explaining species coexistence (Sweson et al., 2006; Yang et al., 2018; Zhang et al., 2010).

At present, the majority of studies on the spatial distributions and associations of related species have been performed on broad‐leaved tree species in tropical (Condit et al., 2000; Guo et al., 2017; Murdjoko et al., 2020), subtropical (Yang et al., 2018), and temperate (Liu et al., 2021; Zhou et al., 2019) forests. Although biological functions differ among genera, our understanding of species coexistence is limited to coniferous species. Thus, in the present study, we studied two congeneric *Pinus* species, which are common pioneer species and often later succeeded by *Quercus* (Broncano et al., 1998; Yu, Wang, et al., 2013), in a 25‐ha warm temperate deciduous broad‐leaved forest. The aims of the present study are to reveal (1) whether co‐occurring *Pinus* species exploit distinct niches or habitats, and (2) whether their habitats or ranges are shifting due to climate change. Specifically, we asked the following research questions and tested associated predictions:

1. Q1 – How do the population structure and distribution pattern of the two *Pinus* species in forest change over different life‐history stages?
  Based on the limited dispersal ability of the species and environmental heterogeneity, we predict that the two *Pinus* species, which belong to different growth types and exhibit different spatial distributions, tend to shift from aggregated distributions to random distributions as they grow from small to large trees with changes in scale.
2. Q2 – Do fierce intraspecific and interspecific competition exist in the two *Pinus* species at three life‐history stages?
  Due to the similarity in resource utilization, we predict that interspecific competition is expected to be stronger. However, due to the limited seed dispersal and the conspecific negative density dependence, intraspecific competition might be stronger in PT than in PA.
3. Q3 – What are the relative contributions of topographic and soil variables to the spatial distributions of the species across the three life‐history stages?
  Considering both life history and physiological traits to define the survival strategies of the two congeneric species, we hypothesize that most *Pinus* species specialize based on topography to support their inherent shade intolerance and strong adaptability.

## 2 MATERIALS AND METHODS

### 2.1 Study region

**FIGURE 1:**
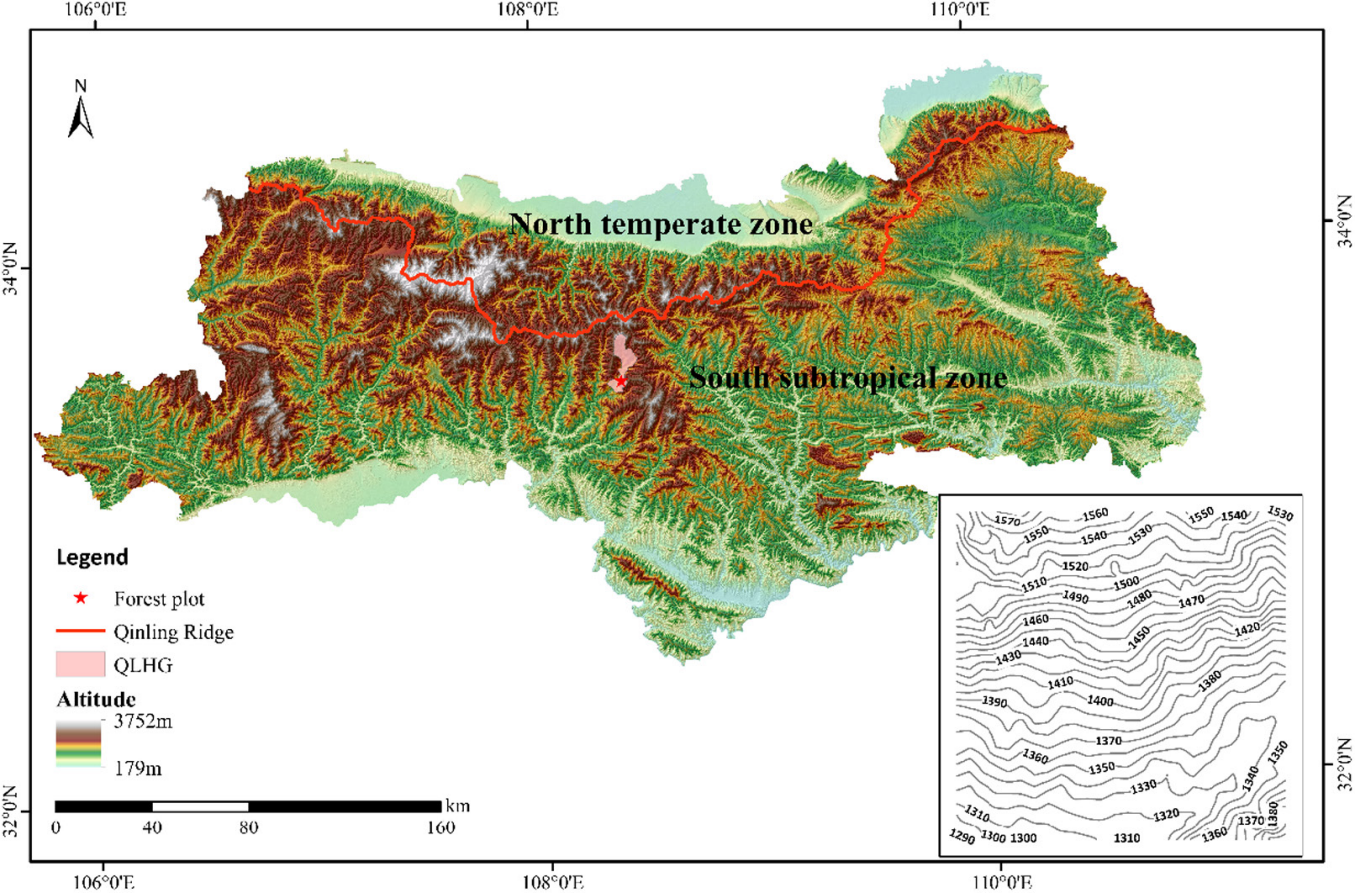
Larger topographic map is represented Qinling Mountains. The red line indicates Qinling ridge, the pink area represents Huangguan town, Qinling, and the plot is represented by the red star. The location and topographic conditions (bottom right) of the 25‐ha Qinling Huangguan Forest dynamics plot (QLHG plot) (pentagram area) as an inset. The numbers in the inset represent elevation (m).

The 25‐ha (500 × 500 m) Qinling Huangguan Forest Dynamics Plot is located on the south slope of the middle section of the Qinling Mountains in a warm temperate deciduous broad‐leaved forest (Figure 1; He et al., 2021; Yin et al., 2019). The plot was established following the standard field protocol of the Chinese Forest Biodiversity Monitoring Network (CForBio) and the Center for Tropical Forest Science (CTFS) (Condit, 1998). The elevation ranged from 1280.3 to 1581.8 m, with an average of 1414.2 m. The first forest inventory was completed in 2019, in which species presence and position were labeled, individual trees were labeled, and diameter at breast height (DBH) was recorded for trees with a DBH ≥1 cm. In total, 75,139 individuals were recorded, belonging to 121 species, 83 genera, and 44 families. The main tree genera in the study area are *Quercus* and *Castanea* (Fagaceae), *Fraxinus* (Oleaceae), *Carpinus* (Betulaceae), and *Pinus* (Pinaceae).

### 2.2 Species investigated

The two focal species PA and PT belong to the genus *Pinus*, family *Pinaceae*, and are widespread in Qinling. They play important ecological roles in conserving nutrients, preventing erosion, and promoting regional socioeconomic development (Critchfield & Little, 1966; Dong et al., 2016; Ning et al., 2021).

**FIGURE 2:**
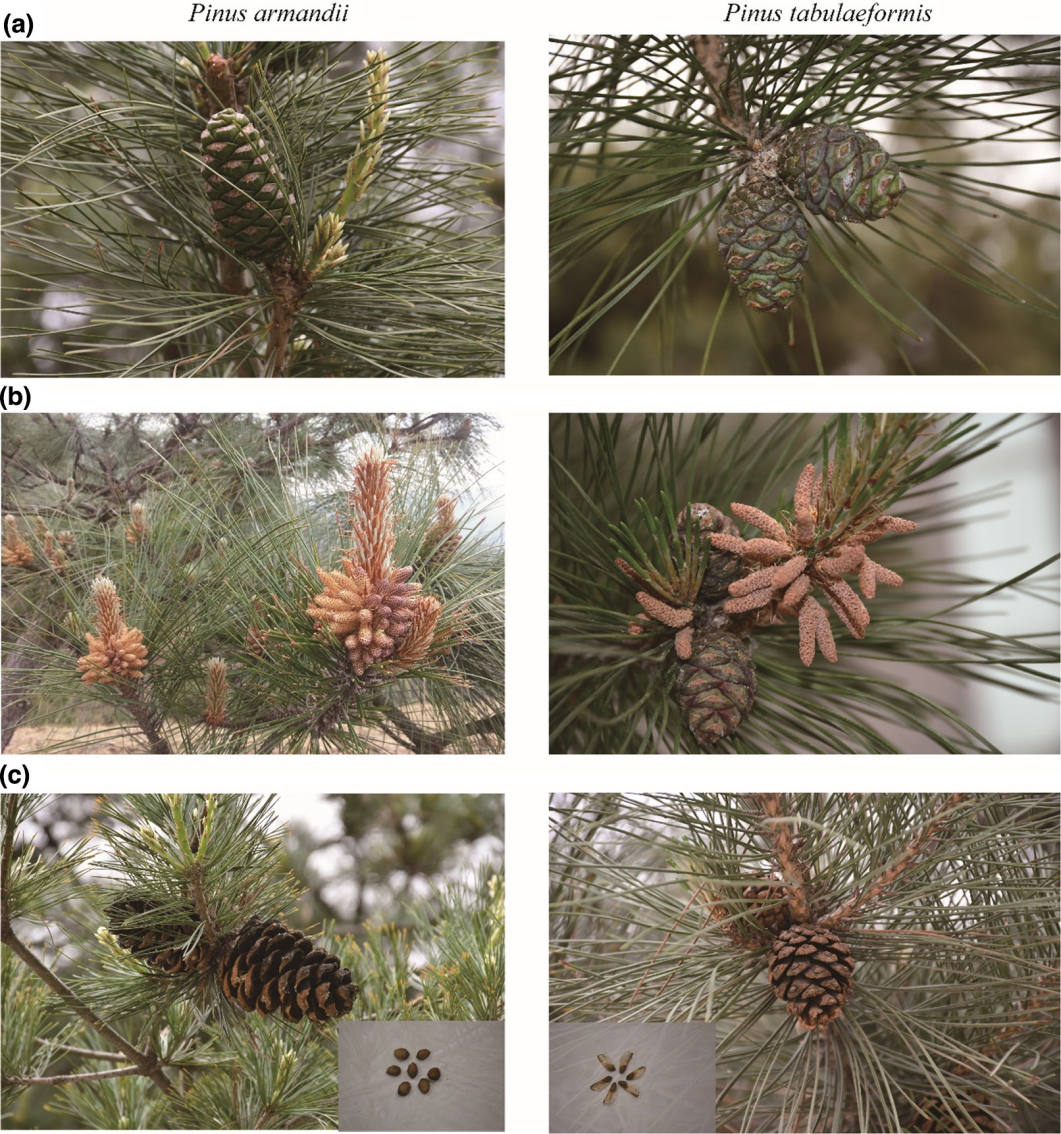
Morphological traits of the two studied Pinus species: i.e. ((a) fruits, (b) anthotaxy, (c) cone and seeds).

In the study plot, *Pinaceae* accounted for 6.5% of the total number of sampled individuals. PT and PA were the two dominant *Pinaceae* species, contributing the most to the structure of the young forest. The remaining *Pinaceae* species (*Tsuga chinensis*) was excluded from this study due to its low abundance (i.e., with only five individuals). There are some differences in morphological characteristics and life history between the two focal *Pinus* species (Huo et al., 2019; Lan et al., 2007). For example, PA seeds are large and wingless, whereas PT seeds are small and membranous‐winged (Figure 2c), and so, this variation causes the species to disperse differently and form different spatial distribution patterns.

### 2.3 Data analysis

**FIGURE 3:**
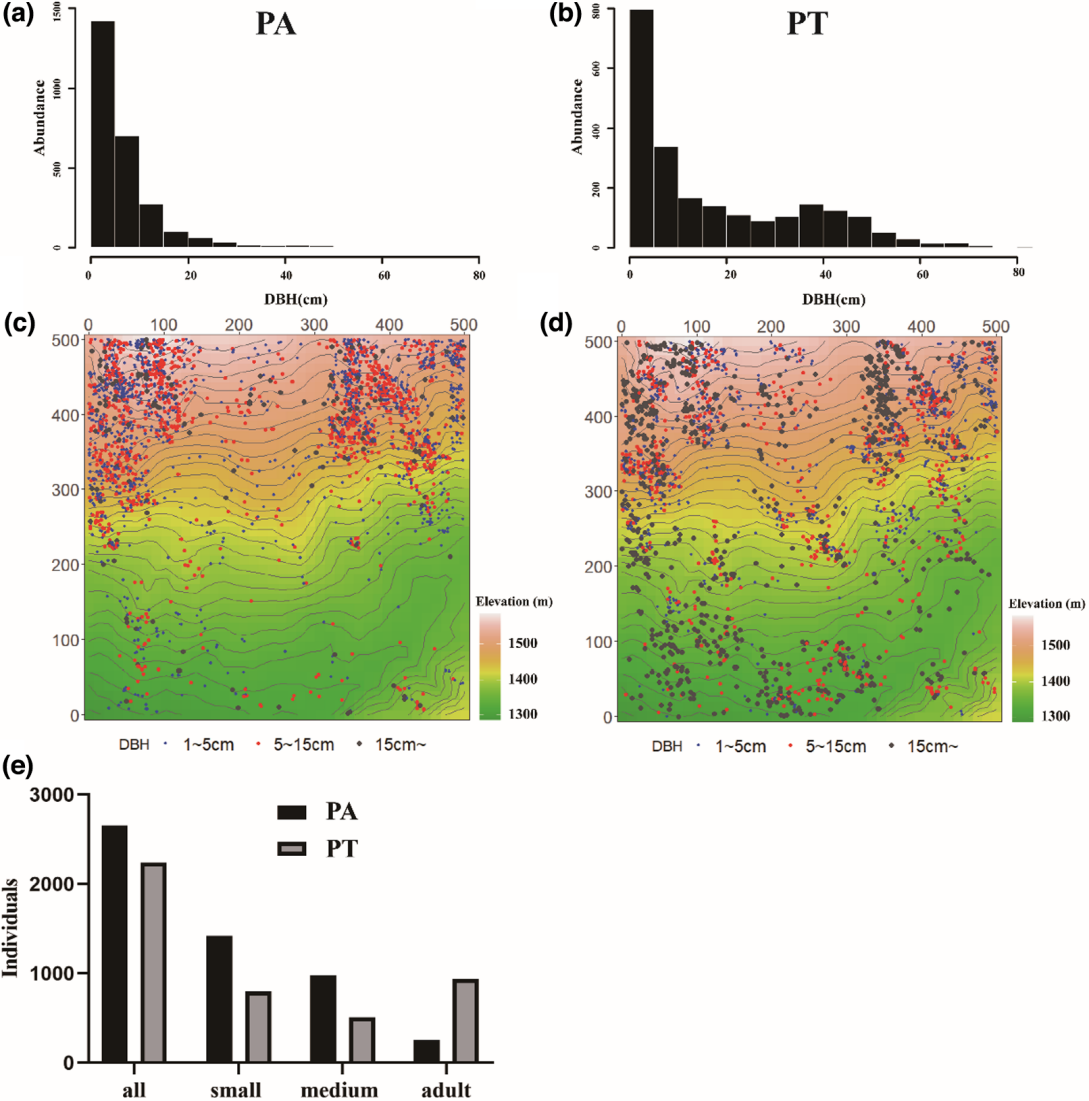
DBH class distribution ((a) PA and (b) PT), spatial distribution ((c) PA and (d) PT) and individual distribution (e) in three developmental stages of the two *Pinus* species (*Pinus armandii* (PA) and *Pinus tabulaeformis* (PT)) in the QLHG plot. Small trees, DBH <5 cm; medium trees, 5 cm ≤ DBH < 15 cm; large trees, DBH ≥15 cm.

In this study, we grouped individual *Pinus* trees into three classes based on DBH: (a) small trees, i.e., 1 cm ≤ DBH <5 cm; (b) medium trees, i.e., 5 cm ≤ DBH < 15 cm; and (c) large trees, i.e., DBH ≥15 cm (Table 1 and Figure 3a,b). We conducted all the following analyses separately for the three size classes of each studied species.

**TABLE 1:** Population structure of two *Pinus* species in a 25‐ha plot established in a warm temperate deciduous broad‐leaved forest in a climate transition zone

Tree species	Species codes	Individuals	Mean DBH	Mean basal area	Importance value	Shade Tolerance	Dispersal mode	Seed size
*Pinus tabuliformis*	PT	2239	17.847 cm	4.41 m^2^/ha	4	Shade	Wind	small
*Pinus armandii*	PA	2653	7.028 cm	0.88 m^2^/ha	7	Intolerant	Animal (rodent)	large

*Note*: importance value = (relative abundance + relative frequency + relative significance)/3; relative abundance = the number of species in the community; relative frequency = ∑Pi _frequency_ / ∑P_i total frequency_; relative significance = ∑P_i breast height area_ / ∑P_i,l breast height area × 100%_.

#### 2.3.1 Analysis 1: Analysis of spatial patterns

To test for similarities in the spatial patterns of congeneric species in the same habitat, we used the pair‐correlation function g(r), which combines univariate and bivariate statistics. The pair‐correlation function g(r) is the derivative of Ripley's popular K function (Anselin & Getis, 1992; Hambly et al., 1994). Compared with Ripley's K function, the pair‐correlation function g(r) eliminates the K function for the large‐scale cumulative effect and can easily distinguish the degree of deviation of the actual distribution of points on a certain scale from the expected value, making it valuable method for analyzing the degree of aggregation (Condit et al., 2000; Wiegand et al., 2004). Here, we first analyzed the spatial distributions of the two *Pinus* species using the univariate g(r) and then analyzed the degree of association using the bivariate g(r), including the intra‐ and interspecific associations of the *Pinus* species at different life‐history stages. To test whether habitat heterogeneity had a significant influence on the spatial distribution of trees, we used the complete spatial randomness (CSR) model, which does not consider spatial heterogeneity, assuming that the spatial distributions of the species were not affected by any biological or nonbiological processes and that all points in the study area had the same probability. We then used the heterogeneous Poisson (HP) model to test for a habitat heterogeneity effect, which can eliminate the influence of large‐scale environmental heterogeneity (Diggle, 2013). For pattern analysis with the univariate statistic, an intensity function was established based on the distributions of the two congeneric species. A second intensity function was built for analysis with the bivariate statistic, where we held the locations of the trees of the first species fixed and randomized the locations of the trees of the second species using the HP model. A bandwidth of 30 m and a spatial resolution of 2 m were chosen for all analyses (Yang et al., 2018; Zhang et al., 2010). To account for asymmetric competition between the two *Pinus* species and between life‐history stages, the species were analyzed as species 1 and species 2, respectively (Getzin et al., 2006).

To compare the strengths of interactions between individuals at different life‐history stages, the bivariate g(r) function random label null model (RL) and a case–control design were used to examine the effects of density restriction on population regulation across life‐history stages. It was assumed that the filtering effect of habitat heterogeneity on trees increases proportionally with developmental life stage, and that the distribution pattern tends to be stable at later stages. According to these assumptions, the distribution pattern of large trees represents the result of habitat heterogeneity. The distribution pattern of large trees in the plot was used as a control (large = pattern 1) to represent the factor of habitat heterogeneity, while the distribution pattern of other trees (small and medium trees) was used as a case pattern (others = pattern 2) (Goreaud & Pélissier, 2003). Here, g_21_(r), g_12_(r) represent the distribution intensity of the control (case) individuals around the cases (control), and g_11_(r) represents the distribution intensity of the control individuals around the control, g_22_(r) represents the distribution intensity of the case individuals around the cases. Using equation (1) (2) to test the statistical analysis of density‐dependent. And (1) d(r) = g_22_(r) − g_11_(r) was used to evaluate the strength of the self‐thinning effect; (2) d(r) = g_21_(r) − g_22_(r) and g_12_(r) − g_22_(r) were used to analyze the density‐dependent thinning effect (Diggle & Chetwynd, 1991; Gatrell et al., 1996; Wiegand et al., 2004).

For all analyses, the Monte‐Carlo simulation was repeated 199 times to yield a 99% confidence interval for each process with the corresponding null model, with a threshold spatial scale of 0–50 m. Points above the upper limits indicate an aggregated distribution or a positive association, points within an intervals indicate a random distribution or a nonsignificant association, and points below the lower limits indicate a regular or negative association (Loosmore & Ford, 2006; Yuan et al., 2018). We used a goodness‐of‐fit test to detect significant departures from the null model and to avoid inflated significance values due to multiple tests across values of r. All point pattern analyses and correlation analyses were performed in Programita software, while GraphPad Prism 8 was used to create graphs.

#### 2.3.2 Analysis 2: Analysis of associated topographic and soil factors

In the forest study plot, three topographic variables were evaluated: i.e., elevation, convexity, and slope. Considering the multicollinearity problem, we use the variance inflation factor (VIF) method to eliminate any factors with overly high VIF values. We chose six soil properties, pH, nitrogen (N), available phosphorus (AP), available potassium (AK), organic matter (OM), and alkali‐hydrolyzable nitrogen (AHN) (Table 2), for measurement in each of the 625 subplots (20 × 20 m). There were 972 soil sampling points, each with a sampling depth of 0–10 cm. Elevation was calculated as the average elevation over the four corners of each subplots (Harms et al., 2001). Convexity was defined as the elevation of a focal quadrat minus the average elevation of the eight surrounding quadrats (Yamakura et al., 1995). In particular, the convexity of edge quadrats was the difference between the elevation at the center point and the average elevation of the four corners. Slope was calculated as the average angle of the four planes deviating from the horizontal plane. The ranges of the three topographic attributes were as follows: elevation, 1292.74–1576.81 m; slope, 3.10°–47.5°; and convexity, −9.15–19.34 m (Table S1). Soil factors were analyzed by the kriging interpolation method (Fu et al., 2020).

**TABLE 2:** Percentage and significance of the interpretation rate of single factors relative to the total interpretation rate of topographic and soil factors for the two *Pinus* species (PA and PT)

Variables	VIF^a^	Unique	Average.shared^b^	Individual importance	I. Perc(%)^c^	*p*‐value^d^
ele	1.58	0.0314	0.0281	0.0595	26.23	.001
slo	1.25	0.0158	0.0096	0.0254	11.2	.001
con	1.27	0.0116	0.0059	0.0175	7.72	.001
PH	8.88	0.0014	0.0232	0.0246	10.85	.001
K	36.4					
P	40.66					
AHN	9.07	0.001	0.0215	0.0225	9.92	.001
AK	8.39	0.0018	0.0284	0.0302	13.32	.001
OM	11.36	0.0044	0.01	0.0144	6.35	.001
AP	13.06	0.0016	0.0158	0.0174	7.67	.001
N	5.09	−0.0009	0.0162	0.0153	6.75	.001
Total		0.0681	0.1587	0.2268	100.01	
Unique to topography					39.69	
Unique to soil					46.39	
Common					13.92	
Total					100	

Abbreviations: AHN, alkali‐hydrolyzable nitrogen; AK, available potassium; AP, available phosphorus; con, convexity; ele, elevation; K, potassium; N, nitrogen; OM, organic matter; P, phosphorus; slo, slope.

^a^ Variance inflation factor

^b^ The total average variance contribution of the explanatory variables.

^c^ Individual effects as a proportion of total corrected *R* ^2^.

^d^ *P‐*value for the permutation test based on 999 randomizations.

To infer the mechanisms by which the environmental factors influenced the two *Pinus* species, redundancy analysis (RDA) was applied to the topographic and soil factor data, and a Monte‐Carlo permutation test was employed to assess the significance of the relationships using the “rdacca.hp” package in R 4.0.1 (Lai et al., 2021). Each topographic or soil factor was tested at the 5% significance level using 999 random permutations. We further used variance partitioning (VP) and hierarchical partitioning (HP) (Lai et al., 2021) analysis to determine the proportion of variation in community structure and different life‐history stages by the specified environmental factors. We combined data for both topography and soil resource variation to investigate the relative contributions of these factors to the spatial distributions of the congeneric species.

## 3 RESULTS

### 3.1 Population structure and spatial patterns

**FIGURE 4:**
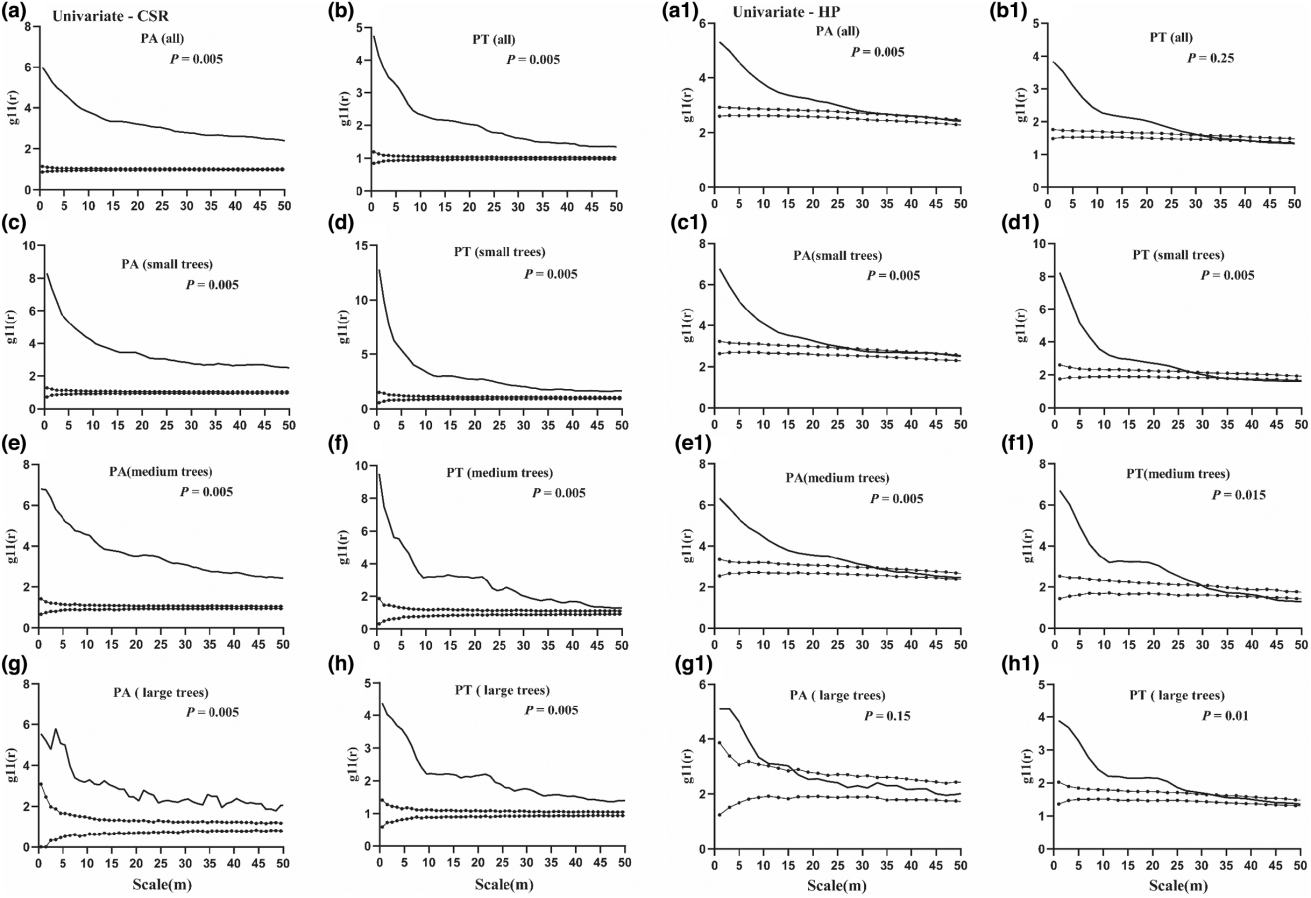
Univariate point pattern analysis of two *Pinus* species (PA and PT) using CSR (a)‐(h) and HP (a1)‐(h1) null models. Black lines indicate the g_11_(r) function; dotted lines indicate the upper and lower limits of the 99% confidence interval. Points above the upper limits indicate an aggregated distribution, those within the intervals indicate a random distribution, and those below the lower limits indicate a regular distribution. The 99% confidence intervals were calculated using the 5 highest and 5 lowest values of g_11_(r) derived from 199 Monte‐Carlo simulations of the heterogeneous Poisson null model.

The numbers of individuals of PT and PA were 2239 and 2653, respectively (Table 1; Figure 3e). The mean basal area and mean DBH of PT were significantly larger than those of PA. Specifically, the DBH distribution of PA was distinctly L‐shaped, whereas bimodal and continuous distributions were observed for PT. In addition, for PA, small trees of were more abundant than large trees, whereas PT showed the opposite pattern; furthermore, the large individuals of PA were less abundant than those of PT. Additionally, PT was distributed over nearly the entire plot, whereas PA was concentrated in the northeast corner and the northwest part of the plot. The spatial distributions of both species showed significant aggregation (Figures 3 and 4).

The overall spatial distributions of the two species were similar (Figure 4a,b), i.e., both showed significant aggregation under the CSR model in the study scale range (Figure 4c–h), whereas much weaker aggregation was observed under the HP model than under the CSR model (Figure 4a1–h1). The HP model excluded habitat heterogeneity at scales over 30 m. According to this null model, the degree of aggregation of the two species decreased with increasing scale, which was consistent with their natural distributions in the plot (Figures 3 and 4). In particular, the spatial distributions of small trees were quite similar between PA and PT (Figure 4c1,d1). In addition, medium trees of PA and PT exhibited high clustering intensity at 0–30 m scales but randomness at >30 m scales (Figure 4e1,f1), whereas the distributions of large trees were dissimilar between PA and PT and exhibited differences among different scales (Figure 4g1,h1). Large individuals of PA exhibited a strong aggregation in the 0–17 m scale range and then a random distribution in the >17 m scale range, while large trees of PT were notably aggregated in the 0–30 m scale range and randomly distributed at >30 m scales (Figure 4g1,h1).

### 3.2 Density dependence

**FIGURE 5:**
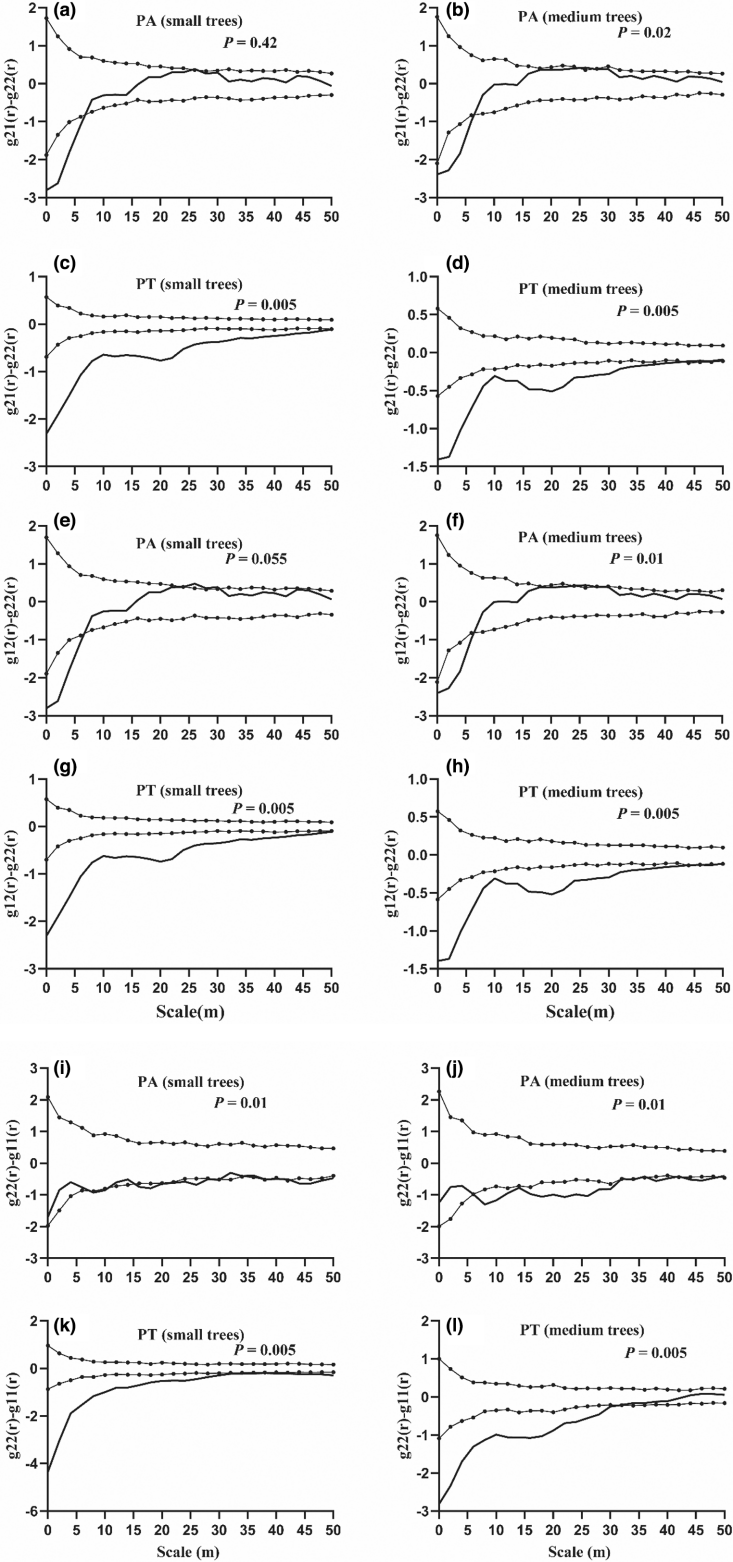
Analysis of intraspecific density‐dependence effects with a case–control design of two *Pinus* species (PA and PT) in the 25‐ha QLHG plot. (a)‐(h) represents thinning effects, (i)‐(l) represents self‐thinning. Black lines indicate observations. Dotted lines indicate the upper and lower limits of the 99% confidence interval. Points above the upper envelope indicate the aggregation of small trees is higher than large trees, points between the envelopes indicate no difference in size aggregation, and points below the lower envelope indicate the aggregation of large trees is higher than small (indicating self‐thinning [or thinning]). The 99% confidence intervals were calculated using the 5 highest and 5 lowest values of 199 Monte‐Carlo simulations of the RL null model.

To explore the potential driving mechanisms of changes in the spatial patterns of the *Pinus* species, we removed habitat heterogeneity through a case–control design and RL null model (Figure 5). For PA, small and medium trees were less aggregated (g_22_(r) − g_11_(r) < 0) than large trees in the 6–50 m scale range (Figure 4i,j). For PT, small trees were less aggregated than large trees at the study scale, and large trees were more aggregated than medium individuals in the 0–30 m scale range (g_22_(r) − g_11_(r) < 0) (Figure 5k,l). In addition, for PA, small (or medium) trees were distributed more frequently around other small (or medium) trees than around large trees in the 0–7 m scale range (Figure 5a,b,e,f), indicating that small trees were less aggregated around large individuals (g_21_(r) − g_22_(r), g_12_(r) − g_22_ (r) < 0). In contrast, for PT, small and medium trees showed significant aggregation around themselves in all studied ranges (g_21_(r) − g_22_(r) and g_12_(r) − g_22_(r) << 0) (Figure 5c,d,g and h). This finding suggested an additional aggregation pattern for small individuals relative to large trees. Small individuals of PA exhibited a weak self‐thinning effect in the 6–50 m scale range and a thinning effect from small to large trees in the 0–7 m scale range; Small individuals of PT exhibited significant self‐thinning and thinning effects from small to large trees in the 0–50 m scale range.

### 3.3 Spatial associations

**FIGURE 6:**
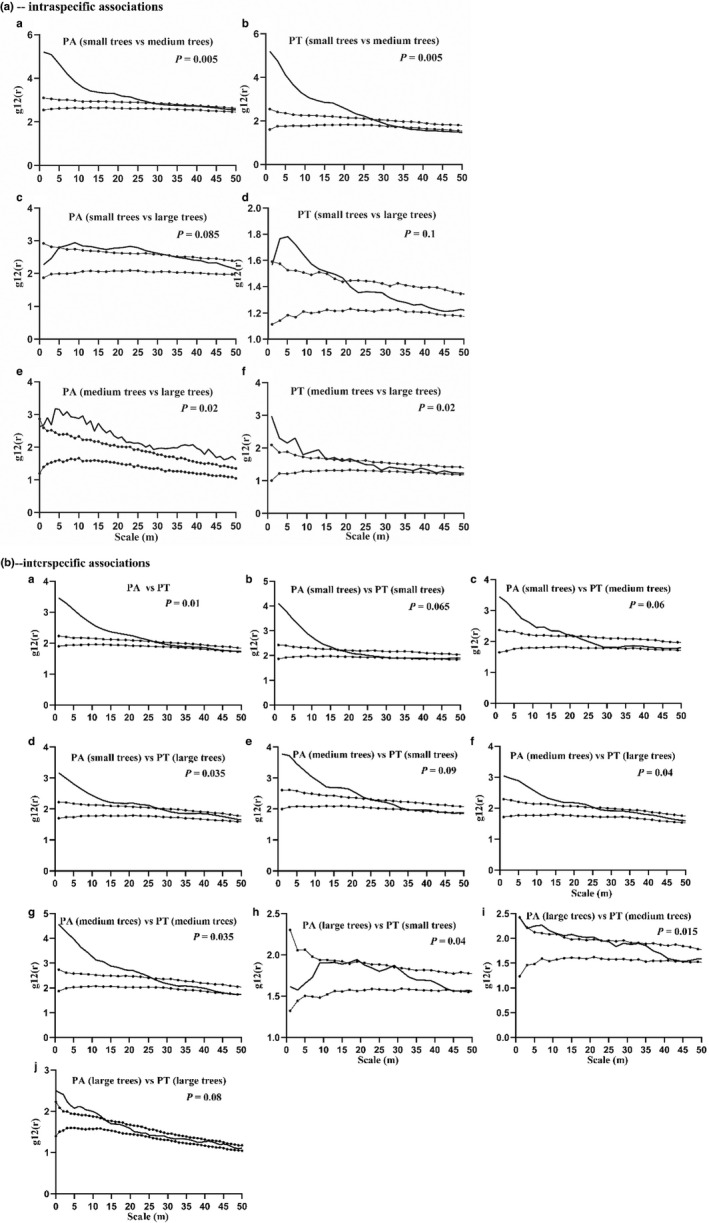
Bivariate point pattern analysis examples for intra‐ and interspecific associations among three size classes of two *Pinus* species (PA and PT). (a) ‐ Intraspecific associations (a‐f), (b) ‐ interspecific associations (a‐j). Black lines indicate the g_12_(r) function; dotted lines indicate the upper and lower limits of the 99% confidence interval. Points above the upper limits indicate positive associations, points within the intervals show nonsignificant associations, and points below the lower limits show negative associations. The 99% confidence intervals were calculated using the five highest and five lowest values of g_12_(r) derived from 199 Monte‐Carlo simulations of the heterogeneous Poisson null model.

We analyzed the cross‐spatial associations between individuals of the two *Pinus* species in the three life‐history stages (Figure 6A). The intraspecific associations were different among the three life‐history stages. Small and medium trees of PA and PT were significantly positively correlated with medium trees in the 0–27 m scale range; small to medium trees of PA were not associated in the 27–50 m scale range; and those of PT showed a shift from no association to a negative association in the 27–50 m scale range (Figure 6A [a,b]). However, small (or medium) individuals of PA showed a nonsignificant positive association with large individuals in the study scale range (Figure 6A[c,e]). Small (medium) individuals of PT showed different degrees of positive association with large individuals in the 0–20 m scale range (Figure 6A[d,f]).

The interspecific association between PA and PT was further tested based on the HP model (Table S2; Figure 6B), and we found positive associations between two pairs of the species in the 0–50 m scale range and positive associations for 16 pairs and no spatial associations for 2 pairs at small scales. Ten of 16 pairs showed positive associations and thus proved symmetrical including PA‐s and PT‐s in the 0–16 m scale range, PA‐s and PT‐m at 0–22 m, PA‐s and PT‐l at 0–26 m, PA‐m and PT‐s at 0–24 m, and PA‐m and PT‐l at 0–22 m. Only three pairs, namely PT‐s vs. PA‐s, PT‐s vs. PA‐m, and PT‐m vs. PA‐s, showed negative associations, all in the 30–40 m scale range.

### 3.4 Contribution of topographic and soil variables to the spatial distributions of *Pinus* species at different life‐history stages

Convexity was more variable than the other environmental variables. The variability of soil N content was greater than that of the other soil variables, and the variabilities of soil AK and OM contents were similar. The soil pH, AHN, AK, and OM levels decreased significantly with increasing elevation. Soil pH and AK were significantly negatively correlated with slope. Moreover, soil K, N, P, AP, and OM were significantly correlated with convexity. PA and PT were mainly distributed in the zones of low‐N, OM, AP, and P in the northern part of the plot (Tables S1 and S3; Figure S1).

**FIGURE 7:**
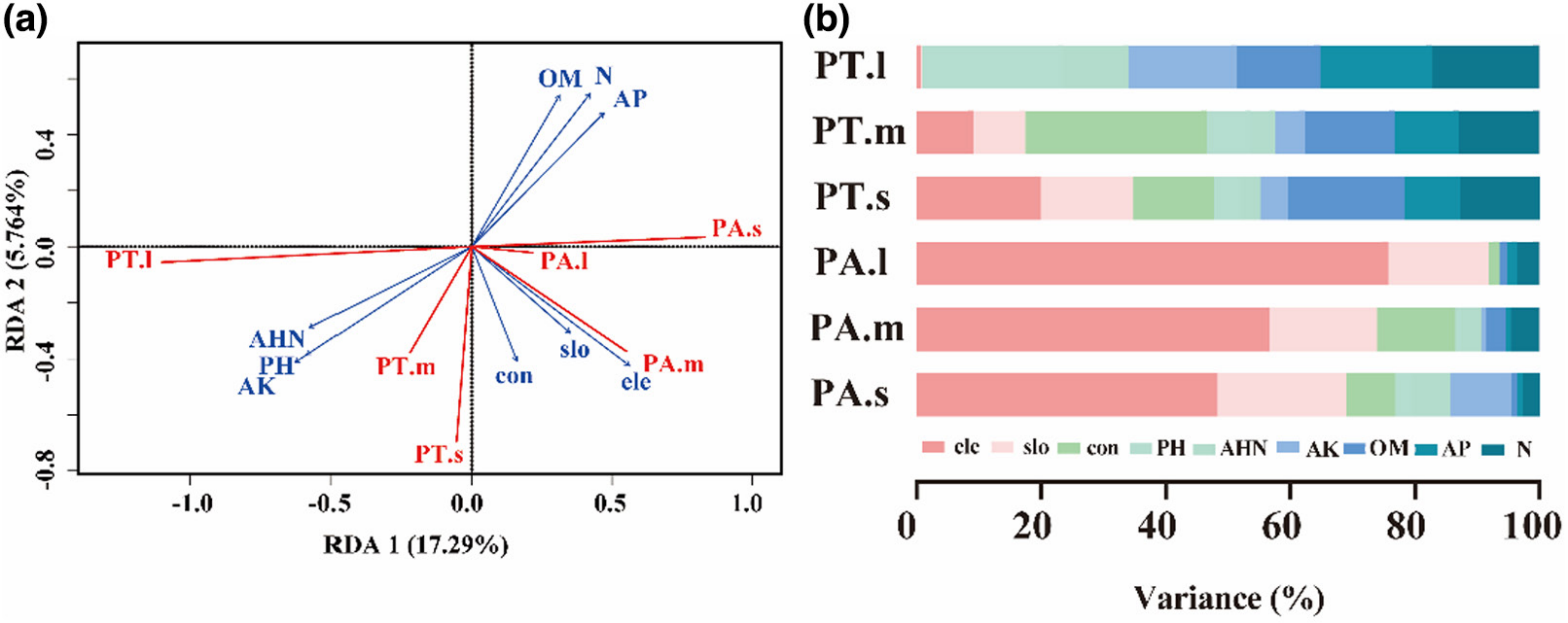
Redundancy analysis (RDA) biplot (a) and variance partitioning and hierarchical partitioning (HP) (b) of the effect of topography and soil factors on the two *Pinus* species (PA and PT) at different life‐history stages. PT.s, PT.m, PT.l, PA.s, PA.m, PA.l represents small trees, mediem trees and large trees of *Pinus tabulaeformis* (PT) and *Pinus armandii* (PA). The environmental factors included elevation (ele), convexity (con), slope (slo), and six soil properties, namely pH, nitrogen (N), available phosphorus (AP), available potassium (AK), organic matter (OM), and alkali‐hydrolyzable nitrogen (AHN).

According to the RDA, the first two axes explained 23.054% of the variation in the species–environment relationships (Figure 7a). The VP and HP results showed that topographic factors alone explained 39.69% of the variance, whereas soil factors alone explained up 46.39%; however, both together explained only 13.92% (Table 2). Elevation was the most important factor overall, followed by slope, soil AK, and pH (Figure 7a,b; Table 2). The responses of the two *Pinus* species to the nine factors at each of the three life‐history stages were studied, and PA was found to be mostly aligned with the first axis of the RDA, i.e., significantly positively correlated with elevation, slope, and convexity significantly correlated with OM, AP, and N. In contrast, small individuals of PT generally aligned with the second axis being significantly positively correlated with elevation, convexity, slope, AK, pH, and AHN. The distributions of large individuals of PT were significantly positively correlated with pH and AK, significantly negatively correlated with OM, AP, and N (Figure 7a). The relative contributions of topographic and soil factors differed among the three life‐history stages (Figure 7b). As predicted, elevation, slope, and convexity were the main contributors to the distribution of PA and were also the major contributors to the distribution of small individuals of PT. Contrary to expectations, pH, AK, OM, AP, and N were the main drivers of the distribution of large trees of PT.

## 4 DISCUSSION

According to the tree abundance and DBH class structure, PA showed better population regeneration structure having more young individuals than PT (Figure 3a,b). Furthermore, a low abundance of small trees of PT was observed, which was likely due to low recruitment rates or high mortality among saplings. Contrary to one of our predictions, the two *Pinus* species exhibited similar spatial distribution patterns, and there was a shift from an aggregated distribution to a random distribution with the transition from small to large trees; thus, significant spatial heterogeneity was observed at small scales (Stephan et al., 2014). Previous studies have shown that the life‐history traits of *Pinus* are associated with their spatial patterns (Jia et al., 2021; Yu et al., 2014), for example, PA and PT are shade‐intolerant species that are tolerant to thinning and seedling shade; these traits contribute to their regeneration in suitably sized gaps. Furthermore, we found that for both PA and PT, the proportion of small trees increased larger with increasing elevation (Figure 3c,d), indicating that the *Pinus* species tend to migrate to higher altitudes (Figure 3). Khalatbari Limaki et al. (2021) reported that the effects of climate change on species distributions usually result in a shift of species to higher altitudes and latitudes, suggesting that environments with much sunshine and exposed soil could be important drivers for the establishment and survival of small trees. However, shrubs and small trees in high‐altitude forests are usually sparse and weakly competitive; accordingly, we found that small trees showed low survival at low altitudes. This result might be attributed to the dense forest canopy or strong interspecific competition and slow metabolism at low altitudes under the context of climate change (Coomes & Allen, 2007).

Recent studies on species coexistence have suggested that conspecific negative density dependence is an important mechanism in regulating plant populations (Piao et al., 2013). By combining intraspecific association and density‐dependent analyses, we found that among PA individuals, small trees were significantly positively associated with medium individuals but only very weakly with large individuals (Figure 6A[a,c]). In PT, small individuals were not significantly correlated with large individuals (Figure 6A[d]), whereas PA exhibited thinning effects and strong self‐thinning effects at small scales (Figure 5a,b,e,f,i,j). However, the thinning and self‐thinning effects of PT were stronger than those of PA (Figure 5c,d,g,h,k,l). As we predicted, conspecific neighbor density had a significant and strong negative impact on survival in the two *Pinus* individual species (Figure 5i–l), which is consistent with the findings of previous studies on density dependence (Uriarte et al., 2004; Wu et al., 2016; Yu, Shi, et al., 2013; Zhu et al., 2010). However, PA was weaker than PT, which is consistent with the finding of Lebrija‐Trejos et al. (2016) that small individuals of species with larger seeds tended to be less negatively impacted by conspecific neighbor than are small individuals of species with smaller seeds. This result further illustrated that the overall pattern of each species was directly determined by the pattern of gap recruitment of small competing trees (Wolf, 2005). This did occur with pioneer *Pinus* species in the QLHG plot, because the canopy gaps were usually large enough for shade‐intolerant small individuals of PA and PT to regenerate underneath. This may be due to the reason that species limited to seed dispersal and the competition for shared resources in the same life‐history stage on a certain scale could have specialized natural enemies (predators and pathogens) (Boege & Marquis, 2005; Liang et al., 2016).

In addition, we found substantial differences among the different life‐history stages of the two species in the effects of conspecific trees; for instance, large seeds of PA had high palatability and more seeds of PA than of PT were eaten or otherwise dispersed by small rodents (Yu et al., 2014). However, in PT, a clustered distribution may facilitate their competition such that a random distribution may be necessary for species coexistence in mixed stands. Our results are consistent with the hypothesis of Jansen 1970 that small seeds are dispersed closer to their parent trees (or seed stations) and thus suffer from strong conspecific negative density dependence. Overall, these results indicate that density dependence differs between species and exhibits wide variation in strength among species (Kobe & Vriesendorp, 2011), and that such interactions between conspecific individuals contribute to species coexistence (Janneke et al., 2002). Therefore, the primary seed dispersal mode plays a significant role in shaping the spatial pattern of *Pinus* species in young forests, which varies among life‐history stages (Kang et al., 2014; Xie et al., 1999).

Closely related congeneric species tend to share many similar phenotypic traits and ecological traits and utilize shared resources in similar ways, making it impossible for them to un‐coexist stably (Mooney et al., 2008; Ribeiro et al., 2021a; Simberloff, 1970). Previous studies have shown strong negative associations between congeneric species with similar ecological properties (Wang et al., 2010). However, contrary to expectations, we found obvious positive interactions between the two pioneer *Pinus* species at a small scale (Figure 6B), at which interspecific competition was weak and existed only among small individuals (Table S2), possibly representing interspecific competition for sunshine. The symmetry of associations (Table S2) indicated that this similarity can strengthen the species' competitive ability and promote local exclusion of heterogeneric species, resulting in a wider ecological niche (Yuan et al., 2018). These similarities of the *Pinus* species did not necessarily cause strong competition between them. In contrast, it might have promoted their stable coexistence. There was almost no significant spatial association between the large PA trees and small PT trees (Figure 6B[H]). Moreover, individuals of the same size class and with the same shade tolerance were not significantly positively associated at small scales, contrast to the findings of Kang et al. (2014, 2017). This inconsistency might be due to the particular height variation and topography of our studied forest plot (He et al., 2021), which included rock exposure and convexity variation (Table S1). In our study, compared with the competition, facilitation, and reciprocity seemed to determine the spatial distributions of the pioneer species in the young forest. Our results are consistent with habitat preference theory (Valiente‐Banuet & Verdú, 2007); i.e., the two co‐occurring congeneric *Pinus* species might have diverged in some key habitat preferences such that they avoid competition by exploiting distinct habitats. Previous studies have shown that habitat specialization may support the coexistence of congeneric species in sympatry (Allié et al., 2015; Itoh et al., 2010; Yamasaki et al., 2013). Ribeiro et al. (2021) found that three congeneric species were spatially independent and exhibited similar habitat preferences in Brazilian white‐sand flooded tropical forests. Our hypotheses need to be further evaluated under the context of environmental influence on the spatial distribution of species.

Topography variations give rise to variances in hydrothermal conditions and can influence the distributions of trees (Baldeck et al., 2013). The complex terrain in the QLHG plot was formed by the elevation variation at the large scale and the exposed rock at the small scale in the warm temperate deciduous broad‐leaved forest (He et al., 2021; Yin et al., 2019), both of which affected the spatial distributions of the two *Pinus* species to different degrees. We found that the relative contributions of topography and soil factors differed among the life‐history stages. Some studies have shown that plant growth is greatly affected by heterogeneity in soil properties (Chai et al., 2016); for example, high soil fertility can promote the growth of trees (Aoyagi et al., 2013). In our study, among the topographic factors, elevation was the main driver of the variation in PA, and the correlation gradually changed with the growth of the species (Figure 7b; Luo et al., 2012). This result may be due to the physiological characteristics of PA: it is resistant to barrenness and can grow at high altitudes or in cracks between stones (Wang et al., 2011). Interestingly, the variation in large PT trees was correlated with soil factors but less with topography, whereas small individuals were strongly affected by topography. These results suggest that individuals of earlier developmental stages tend to establish, depending on elevation and slope, for efficient light capture in the shaded understory. At later life‐history stages, individuals no longer need to compete for light but require sufficient nutrients to improve light interception. Furthermore, PA and PT were widely distributed on poor soil, i.e., soil with low fertility, possibly because the litter under mature *Pinus* trees (Briggs et al., 2009) tends to contain tannins and resinous substances that are difficult to decompose and may create a special soil environment (Wan, 2019). Our results confirm that the coexistence stability of the two *Pinus* species is dependent on some key habitats preferences.

Natural selection may cause congeneric species to develop different but mutually beneficial lifestyles, resulting in, for example, mutual attraction for the formation of pine oak mixed forest in the Qinling Mountains (Li et al., 2014; Queenborough et al., 2007). It also reveals the mechanisms for population regeneration and maintenance (Chesson, 2000b; Yang et al., 2012; Yang et al., 2018). However, a large proportion of the variation in the two *Pinus* populations was spatially structured and unaccounted for by the studied soil and topographic variables. Species responses to environmental variables or traits not taken into account in this study (such as functional traits related to resource use strategies, light, soil moisture, and mycorrhizal networks) may explain the remaining portion of unexplained variance (Baldeck et al., 2013). Therefore, it is important to assess how photosynthesis and related leaf traits that are indispensable for tree growth and survival respond to environmental factors, such as elevation and light conditions (Suzuki & Takahashi, 2020). There is evidence that congeneric species can coexist if traits have diverged within the genus (Beltrán et al., 2012), which could be another reason why *Pinus* species can coexist, but further studies are needed to evaluate this possibility.

## 5 CONCLUSIONS

Overall, our results demonstrate how life‐history stage interacts with conspecific density dependence and habitat preference to influence species coexistence in two *Pinus* species. Our findings support the idea that conspecific density dependence and habitat preference contribute to species coexistence. The effects of conspecific negative density dependence of *Pinus tabulaeformis* were stronger than *Pinus armandii*, indicating that small individuals of species with larger seeds tended to be less negatively impacted by conspecific neighbor than are small individuals of species with smaller seeds. Furthermore, our study shows that interspecific competition between the two *Pinus* species was weak and that stable coexistence was likely possible due to distinct habitat preference: *Pinus armandii* was very sensitive to elevation, whereas small individuals of *Pinus tabulaeformis* were sensitive to topography, but large trees were sensitive to soil resource availability. However, small trees of the two *Pinus* species tended to be distributed at higher altitudes, and several small trees were found dead at low altitudes during the forest inventory; these findings might be due to climate change, a possibility that needs further investigation.

## AUTHOR CONTRIBUTIONS

**Zhichun Yang:** Data curation (equal); investigation (equal); software (lead); writing – original draft (lead). **Ying Luo:** Software (supporting); supervision (supporting); writing – review and editing (equal). **Nan Ye:** Software (equal); writing – review and editing (supporting). **Lishunan Yang:** Writing – review and editing (supporting). **Qiulong Yin:** Writing – review and editing (supporting). **Shihong Jia:** Resources (supporting); writing – review and editing (supporting). **Chunmei He:** Investigation (equal). **Zuoqiang Yuan:** Resources (supporting). **Zhanqing Hao:** Resources (lead); supervision (supporting); writing – review and editing (supporting). **Arshad Ali:** Writing – review and editing (supporting).

## CONFLICT OF INTEREST

We declare that we do not have any commercial or associative interest that represents a conflict of interest in connection with the work submitted.

## Supporting information


Appendix S1

## Data Availability

The address of data. https://doi.org/10.6084/m9.figshare.20446017.v1.
