# Supplementary material for: Disentangling the effects of species interactions and environmental factors on the spatial pattern and coexistence of two congeneric Pinus species in a transitional climatic zone
Source: Ecol Evol. 2022 Sep 11;12(9):e9275. doi: 10.1002/ece3.9275 (PMC9465400; doi:10.1002/ece3.9275)
Supplement: Supplementary file 1 — Appendix S1 [file ECE3-12-e9275-s001.docx]

**Appendix:**

Table S1 Descriptive statistics of topographic and soil factors in the 25-ha Qinling Huangguan Forest Dynamics Plot (QLHG plot).

| **Factors** | **Min** | **Max** | **Mean** | **SD** | **CV** |
| --- | --- | --- | --- | --- | --- |
| elevation(ele) (m) | 1292.74 | 1576.81 | 141856 | 76.02 | 0.05 |
| slope (slo) (°) | 3.1 | 47.5 | 27.98 | 8.14 | 0.29 |
| convexity(con) (m) | -9.15 | 19.34 | 0.09 | 3.03 | 33.11 |
| pH | 4.83 | 7.37 | 5.94 | 0.51 | 0.09 |
| nitrogen (N) (g/kg) | 0.3 | 24.89 | 3.5 | 2.88 | 0.82 |
| phosphorus (P) (g/kg) | 0.16 | 1.69 | 0.85 | 0.28 | 0.33 |
| available phosphorus (AP) (mg/kg) | 0.1 | 97.5 | 17.24 | 10.63 | 0.62 |
| available potassium (AK) (mg/kg) | 7.37 | 422.93 | 70.44 | 51.84 | 0.74 |
| organic matter (OM) (g/kg) | 7.37 | 422.93 | 70.44 | 51.84 | 0.74 |
| potassium (K) (g/kg) | 11.68 | 21.86 | 16.54 | 1.42 | 0.08 |
| alkali-hydrolyzable nitrogen (AHN) (mg/kg) | 12.6 | 478.8 | 107.3 | 67.78 | 0.63 |

Table S2 Bivariate point pattern analysis of the spatial associations of two *Pinus* species in the study plot.

| Species A | Species B | *P* value | Scales(m) | | |  | |  | |  | |  | |  | |  | |  | |  | |  | |  | |  | |  | |  | |  | |  | |  | |  | |  | |  | |  | |
| --- | --- | --- | --- | --- | --- | --- | --- | --- | --- | --- | --- | --- | --- | --- | --- | --- | --- | --- | --- | --- | --- | --- | --- | --- | --- | --- | --- | --- | --- | --- | --- | --- | --- | --- | --- | --- | --- | --- | --- | --- | --- | --- | --- | --- | --- |
| interspecies | |  | | 0 | 2 | | 4 | | 6 | | 8 | | 10 | | 12 | | 14 | | 16 | | 18 | | 20 | | 22 | | 24 | | 26 | | 28 | | 30 | | 32 | | 34 | | 36 | | 38 | | 40 | |  |
| PA | PT | 0.01 | ﹢ | | | ﹢ | | ﹢ | | ﹢ | | ﹢ | | ﹢ | | ﹢ | | ﹢ | | ﹢ | | ﹢ | | ﹢ | | ﹢ | | ﹢ | | r | | r | | r | | r | | r | | r | | r | | r | |
| PT | PA | 0.005 | ﹢ | | | ﹢ | | ﹢ | | ﹢ | | ﹢ | | ﹢ | | r | | ﹢ | | ﹢ | | ﹢ | | ﹢ | | ﹢ | | ﹢ | | ﹢ | | r | | r | | r | | r | | r | | r | | r | |
| PA-s | PT-s | 0.085 | ﹢ | | | ﹢ | | ﹢ | | ﹢ | | ﹢ | | ﹢ | | ﹢ | | ﹢ | | ﹢ | | r | | r | | r | | r | | r | | r | | r | | ﹣ | | ﹣ | | r | | r | | r | |
| PA-s | PT-m | 0.085 | ﹢ | | | ﹢ | | ﹢ | | ﹢ | | ﹢ | | ﹢ | | ﹢ | | ﹢ | | ﹢ | | ﹢ | | ﹢ | | ﹢ | | r | | r | | r | | r | | r | | r | | r | | r | | r | |
| PA-s | PT-l | 0.07 | ﹢ | | | ﹢ | | ﹢ | | ﹢ | | ﹢ | | ﹢ | | ﹢ | | ﹢ | | ﹢ | | ﹢ | | ﹢ | | ﹢ | | ﹢ | | ﹢ | | r | | r | | r | | r | | r | | r | | r | |
| PA-m | PT-s | 0.035 | ﹢ | | | ﹢ | | ﹢ | | ﹢ | | ﹢ | | ﹢ | | ﹢ | | ﹢ | | ﹢ | | ﹢ | | ﹢ | | ﹢ | | ﹢ | | r | | r | | r | | r | | r | | r | | r | | r | |
| PA-m | PT-m | 0.02 | ﹢ | | | ﹢ | | ﹢ | | ﹢ | | ﹢ | | ﹢ | | ﹢ | | ﹢ | | ﹢ | | ﹢ | | ﹢ | | ﹢ | | ﹢ | | r | | r | | r | | r | | r | | r | | r | | r | |
| PA-m | PT-l | 0.005 | ﹢ | | | ﹢ | | ﹢ | | ﹢ | | ﹢ | | ﹢ | | ﹢ | | ﹢ | | ﹢ | | ﹢ | | ﹢ | | ﹢ | | ﹢ | | r | | r | | ﹢ | | r | | r | | r | | r | | r | |
| PA-l | PT-s | 0.21 | r | | | r | | r | | r | | r | | r | | r | | r | | r | | ﹢ | | ﹢ | | r | | r | | r | | r | | ﹢ | | r | | r | | r | | r | | r | |
| PA-l | PT-m | 0.065 | r | | | r | | r | | ﹢ | | ﹢ | | ﹢ | | ﹢ | | ﹢ | | ﹢ | | ﹢ | | ﹢ | | ﹢ | | r | | r | | r | | r | | r | | r | | r | | r | | r | |
| PA-l | PT-l | 0.02 | ﹢ | | | ﹢ | | ﹢ | | ﹢ | | ﹢ | | ﹢ | | ﹢ | | r | | r | | r | | r | | r | | r | | r | | r | | r | | r | | r | | r | | r | | r | |
| PT-s | PA-s | 0.005 | ﹢ | | | ﹢ | | ﹢ | | ﹢ | | ﹢ | | ﹢ | | ﹢ | | ﹢ | | ﹢ | | ﹢ | | r | | r | | r | | r | | r | | ﹣ | | ﹣ | | ﹣ | | ﹣ | | ﹣ | | ﹣ | |
| PT-s | PA-m | 0.015 | ﹢ | | | ﹢ | | ﹢ | | ﹢ | | ﹢ | | ﹢ | | ﹢ | | ﹢ | | ﹢ | | ﹢ | | ﹢ | | ﹢ | | ﹢ | | r | | r | | r | | r | | ﹣ | | ﹣ | | ﹣ | | ﹣ | |
| PT-s | PA-l | 0.005 | ﹢ | | | ﹢ | | ﹢ | | ﹢ | | ﹢ | | ﹢ | | ﹢ | | ﹢ | | ﹢ | | ﹢ | | ﹢ | | ﹢ | | ﹢ | | ﹢ | | ﹢ | | ﹢ | | ﹢ | | ﹢ | | ﹢ | | ﹢ | | ﹢ | |
| PT-m | PA-s | 0.005 | ﹢ | | | ﹢ | | ﹢ | | ﹢ | | ﹢ | | ﹢ | | ﹢ | | ﹢ | | ﹢ | | ﹢ | | ﹢ | | r | | r | | r | | r | | ﹣ | | ﹣ | | ﹣ | | ﹣ | | r | | r | |
| PT-m | PA-m | 0.005 | ﹢ | | | ﹢ | | ﹢ | | ﹢ | | ﹢ | | ﹢ | | ﹢ | | ﹢ | | ﹢ | | ﹢ | | ﹢ | | ﹢ | | ﹢ | | ﹢ | | ﹢ | | r | | r | | r | | r | | r | | r | |
| PT-m | PA-l | 0.005 | ﹢ | | | ﹢ | | ﹢ | | ﹢ | | ﹢ | | ﹢ | | ﹢ | | ﹢ | | ﹢ | | ﹢ | | ﹢ | | ﹢ | | ﹢ | | ﹢ | | ﹢ | | ﹢ | | ﹢ | | ﹢ | | ﹢ | | ﹢ | | ﹢ | |
| PT-l | PA-s | 0.005 | ﹢ | | | ﹢ | | ﹢ | | ﹢ | | ﹢ | | ﹢ | | ﹢ | | ﹢ | | ﹢ | | ﹢ | | ﹢ | | ﹢ | | ﹢ | | ﹢ | | ﹢ | | r | | r | | r | | r | | r | | r | |
| PT-l | PA-m | 0.005 | ﹢ | | | ﹢ | | ﹢ | | ﹢ | | ﹢ | | ﹢ | | ﹢ | | ﹢ | | ﹢ | | ﹢ | | ﹢ | | ﹢ | | r | | r | | r | | r | | r | | r | | r | | r | | r | |
| PT-l | PA-l | 0.005 | ﹢ | | | ﹢ | | ﹢ | | ﹢ | | r | | r | | r | | ﹢ | | ﹢ | | ﹢ | | ﹢ | | ﹢ | | ﹢ | | ﹢ | | ﹢ | | ﹢ | | ﹢ | | ﹢ | | ﹢ | | ﹢ | | ﹢ | |

The intensity function was constructed based on the pattern of species B with the bivariate statistic under the heterogeneous Poisson null model with a bandwidth h = 30 m. The ring width for estimation of the pair-correlation function was 4 m; the cell size was 2 × 2 m; and “﹢” means a positive correlation, “r” means no correlation, and “-“ means a negative correlation. Scale = 0, and the points are within the cells. Monte Carlo confidence was inferred at the 99% confidence level (199 simulations). The *p* values were derived from the goodness-of-fit (GOF) test. Only interspecies pairs are shown in the table (s-small trees, m-medium trees and l-large trees). PT-s, PT-m, PT-l, PA-s, PA-m, PA-l represents small trees, medium trees and large trees of *Pinus tabulaeformis* (PT) and *Pinus armandii* (PA).

Table S3 Pearson’s correlations between topographic factors and soil factors (0.95 confidence interval).

| **Factors** | **ele** | | **slo** | | **con** |  |
| --- | --- | --- | --- | --- | --- | --- |
|  | R | P | R | P | R | P |
| pH | -0.2 | <0.001^***^ | -0.12 | 0.0086^**^ | 0.042 | 0.4 |
| K | -0.054 | 0.2 | -0.074 | 0.1 | 0.091 | 0.048^*^ |
| P | 0.04 | 0.4 | 0.0089 | 0.8 | -0.09 | 0.05^*^ |
| AHN | -0.24 | <0.001^***^ | -0.082 | 0.1 | 0.054 | 0.2 |
| AK | -0.2 | <0.001^***^ | -0.13 | 0.0064^**^ | 0.078 | 0.1 |
| OM | -0.13 | 0.0041^**^ | -0.032 | 0.5 | -0.085 | 0.05^*^ |
| AP | -0.26 | 0.6 | 0.018 | 0.7 | -0.13 | 0.0045^**^ |
| N | -0.059 | 0.2 | 0.042 | 0.4 | -0.15 | <0.001^***^ |

Note, ^*^*P* < 0.05, ^**^*P* < 0.01, ^***^*P* < 0.001. Factors, i.e., elevation (ele), slope (slo), convexity (con), pH, nitrogen (N) (g/kg), phosphorus (P) (g/kg), available phosphorus (AP) (mg/kg), available potassium (AK) (mg/kg), organic matter (OM) (g/kg), potassium (K) (g/kg), alkali-hydrolyzable nitrogen (AHN) (mg/kg).


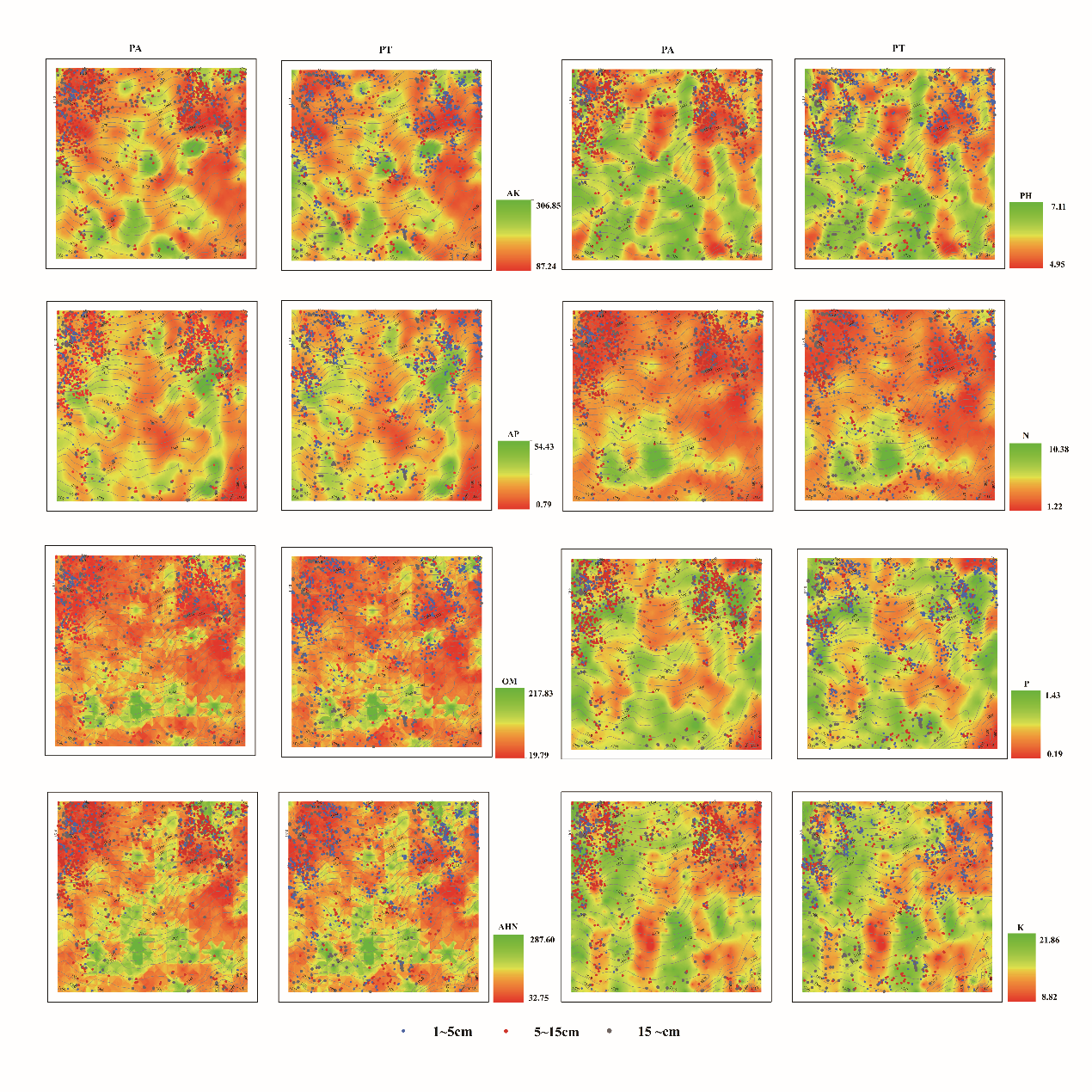


Fig. S1 Spatial distribution of two *Pinus* species in relation to the topography and soil factors (pH, nitrogen (N) (g/kg), phosphorus (P) (g/kg), available phosphorus (AP) (mg/kg), available potassium (AK) (mg/kg), organic matter (OM) (g/kg), potassium (K) (g/kg), alkali-hydrolyzable nitrogen (AHN) (mg/kg)) in the QLHG plot.
